# Atrial fibrillation risk factor management with a plant‐based diet: A review

**DOI:** 10.1002/joa3.12254

**Published:** 2019-11-06

**Authors:** Maximilian Andreas Storz, Paul Helle

**Affiliations:** ^1^ Department of Intensive Care Medicine Die Filderklinik Filderstadt‐Bonlanden Germany

**Keywords:** atrial fibrillation, lifestyle intervention, plant‐based diet, risk factor, vegan

## Abstract

Atrial fibrillation is the most prevalent cardiac arrhythmia in the clinical setting affecting approximately 34 million individuals worldwide. The disease is associated with a significant burden of morbidity and mortality resulting from stroke, heart failure, and acute coronary syndrome. Atrial fibrillation is now a major public health problem with tremendous implications on the economy and the world's healthcare systems. Numerous risk factors and clinical conditions that are associated with the development and progression of atrial fibrillation have been identified in the past. Within the last decades, a shift in awareness toward modifiable conditions has been observed and risk factor management has gained significant momentum. In light of this, dietary approaches are of paramount importance. Whole‐food plant‐based diets emphasizing grains, legumes, vegetables, fruits and nuts and excluding most (or all) animal products have recently experienced a significantly increased interest. The purpose of this review is to present evidence suggestive of a plant‐based diet being a valuable tool in atrial fibrillation risk factor management. The effects of a plant‐based diet on both established and emerging risk factors, such as inflammation and subclinical atherosclerosis, are reviewed in this article. A special focus is put on cardiovascular and metabolic risk factors including hypertension, coronary artery disease, diabetes, and obesity.

## 1 INTRODUCTION

Atrial fibrillation (AF) is the most common arrhythmia in the clinical setting affecting an estimated 2.7 million to 6.1 million Americans and nearly 33.5 million individuals worldwide.^1^, ^2^ The disease is recognized as a global public health problem due to its significant burden of mortality and morbidity resulting from stroke, acute coronary syndrome, and heart failure.^2^, ^3^ Moreover, AF has vast implications on the economy and public health system. The incremental cost related to AF in the United States is estimated to range from $6.0 to $26.0 billion per year.^3^, ^4^ This burden is expected to further increase within the next decades, illustrating, once again, the need for better primary prevention and AF treatment.^5^, ^6^

Numerous risk factors, such as hyperthyroidism, diabetes, or hypertension are associated with the disease. Targeting these risk factors and other underlying conditions is an emerging AF management concept that warrants better implementation in daily clinical practice.^7^

Several of these risk factors are affected by modifiable lifestyle factors, which lead to the idea that certain lifestyle choices, such as diet, may have a tremendous impact on AF risk and mortality. Thus, the purpose of this narrative review is to present selected evidence suggestive of a plant‐based diet being a valuable tool in modifying these risk factors.

## 2 EPIDEMIOLOGY AND ETIOLOGY

AF burden has significant regional variations, with high‐income countries experiencing a higher prevalence and incidence than low‐ and middle‐income countries.^2^, ^8^ The disease currently affects up to 3% of Western populations aged 20 years or older.^7^ In 2014, Chugh and colleagues compared the prevalence and incidence of AF in Asia and North America.^2^ The authors reported on significantly lower AF incidence rates in the Asia‐Pacific region compared to North America (33.8 vs 264.5 per 100 000 person‐years for men and 19.8 vs 196.3 per 100 000 person‐years for women respectively). It is noteworthy that a large variation in AF prevalence can be seen between studies and countries.^9^, ^10^ Several reviews emphasized a great AF variability existing especially in Asian countries and called for further global epidemiological studies to allow for a more accurate picture of the current AF incidence and prevalence.^10^ While it is predicted that more than 1 million people in Japan will be affected by the disease by 2050,^11^ it is now widely accepted that the AF prevalence is generally lower in Asia than in Europe or the United States.

While genetics, natural aging of the population and reporting patterns play an important role, they may not sufficiently explain this unequal distribution. It is more likely that the accumulation of chronic cardiovascular diseases and other risk factors is a major driver of this growing epidemic.^7^ In this context, a study by Dewland and colleagues revealed that racial differences in AF risk are significantly reduced with the accumulation of cardiovascular comorbidities.^12^

Multiple risk factors and clinical conditions that are associated with the development and progression of AF have been identified in the last decades.^7^, ^13^ Although there are several nonmodifiable risk factors, such as gender or advancing age, a gradual shift in awareness toward modifiable predisposing conditions has been observed.^14^ Conventional cardiovascular risk factors that are associated with AF include hypertension, coronary heart disease, heart failure (both with reserved and preserved ejection fraction), and valvular heart disease.^13^, ^15^ Other well‐established concomitant risk factors include diabetes, overweight, obesity, and hyperthyroidism. Additionally, there is a number of emerging and less well‐researched risk factors, such as subclinical atherosclerosis, inflammation, obstructive sleep apnea, and chronic kidney disease. Finally, there are several dietary components that have been postulated to play a role in the pathogenesis and treatment of AF.^16^

## 3 DIET AND ATRIAL FIBRILLATION

Whether dietary factors predispose individuals to AF is partly unexplored and studies from the past often revealed conflicting results.^17^ At various stages, attention has turned to caffeine, alcohol, fish‐derived long‐chain polyunsaturated fatty acids, and dietary fiber.^16^, ^17^

Several prospective studies have described an association between higher levels of alcohol intake and incident AF.^7^, ^14^ In fact, acute heavy alcohol consumption and binge drinking have been related to AF long ago.^17^, ^18^ An analysis from the Framingham Heart Study revealed a significant association between moderate‐to‐heavy alcohol intake (~ 36 g alcohol/ ≥3 drinks per day) and increased AF risk in men.^17^, ^19^ Moreover, Larsson and colleagues described a statistically significant 8% increase in the relative risk of AF with each standard drink per day.^20^ The precise mechanism by which alcohol intake increases the propensity toward atrial arrhythmias remains unclear.^14^, ^21^ Proarrhythmic effects of alcohol intake include shortening of the atrial refractory period,^21^ diminished vagal and augmented sympathetic heart rate modulation,^22^ and alteration in atrial current densities.^14^

The role of caffeine from coffee, tea, and caffeinated soda on AF is less clear.^17^ Considered a neurohormonal stimulant, caffeine had long been postulated to be proarrhythmic by sympathetic activation.^17^, ^23^ Nevertheless, several more recent cohort studies have failed to show this association between caffeine intake and AF.^17^, ^24^, ^25^ On the contrary, a review by Caldeira et al suggested that low‐dose caffeine may even have a protective effect.^25^

In 2004, the authors of a prospective, population‐based cohort study reported a beneficial association between AF and the intake of fish and fish‐derived polyunsaturated fatty acids.^26^ Other studies, however, could not confirm these findings.^27^, ^28^ Results from the Framingham Heart Study revealed an even increased risk of developing AF in individuals who consumed more than four servings of dark fish per week.^17^ The authors of this study concluded that their observations may even “suggest a true adverse effect” of fish oil and dark fish on certain subtypes of AF. It has been suggested that methyl mercury and other potential toxins accumulated in fish potentially have a negative effect on cardiac arrhythmia.^17^

Finally, low serum magnesium levels have been moderately associated with the development of AF in individuals without cardiovascular disease.^29^, ^30^ Magnesium administration has been shown to be an effective prophylactic measure for the prevention of postoperative AF.^31^ Furthermore, intravenous magnesium administration also seems to be effective in the acute management of rapid AF.^32^ The reservation must be made, however, that another study found no association between dietary magnesium and AF risk.^30^

In short, there is persuasive evidence that avoidance of certain foods (such as dark fish and alcohol) and intake of specific nutrients (such as magnesium) may beneficially affect AF. This ultimately leads to the question of specific dietary patterns targeting risk factors and concomitant diseases predisposing individuals to AF.

## 4 RISK FACTOR MODIFICATION THROUGH DIETARY MODIFICATION

The research on dietary patterns and their relation to AF risk is limited to some extent, however, more recently, risk factor management has gained significant momentum.^14^ Targeting underlying predisposing conditions that are associated with the development and progression of AF seems prudent.^7^ In this context, nutritional approaches are of paramount importance.

A great example is a recent study by Pathak and colleagues, which revealed that weight loss ≥10% in patients suffering from AF resulted in a sixfold greater probability of arrhythmia‐free survival compared to patients who lost less than 10% of their body weight in the same period.^33^ The same study also associated sustained weight loss with significant maintenance of sinus rhythm.

Within the last decades, plant‐based nutrition has experienced increased interest in the medical community. Plant‐based diets may reduce the likelihood of many traditional risk factors that are associated with AF, including hypertension, hyperthyroidism, obesity, and diabetes.^34^ Moreover, plant‐based nutrition may also positively affect emerging risk factors, such as inflammation or subclinical atherosclerosis. Current evidence supporting these statements is present below and in Table 1.

**Table 1:** Atrial fibrillation risk factor modification and plant‐based diets: an overview

Risk factors associated with AF	Association with a plant‐based diet	Suggested mechanisms
Hypertension^7^, ^14^, ^35^	Reduced risk and prevalence of hypertension.^38^, ^39^, ^41^	Improved vasodilation^33^, ^37^, ^38^, increased potassium intake,^40^, ^46^ decreased blood viscosity.^38^, ^47^
Diabetes^7^, ^14^, ^48^	Improved glycemic control and reduced insulin resistance.^51^, ^55^, ^56^	Low content of advanced glycation endproducts and low saturated fat proportion, decreased lipotoxicity.^54^, ^57^, ^58^
Obesity and obstructive sleep apnea^7^, ^14^, ^48^	Reduced risk and prevalence of obesity, suitable diet for weight loss.^59^, ^60^, ^61^	High fiber and low fat content of plant‐based nutrition. Reduced energy density, increased postprandial energy expenditure^60^
Systemic Inflammation 13, 70, 71	Reduces mediators of the inflammatory response (hsCRP).^77^	High in anti‐inflammatory and anti‐oxidative components.^40^, ^73^ Absence of pro‐inflammatory fats.^43^
Coronary Artery Disease^7^	Prevention and even reversal of atherosclerosis and CAD events.^81^, ^82^, ^84^	Prevention of vascular endothelial cell injury.^78^, ^84^ Prevention of LDL oxidation and oxidative inflammation by increased antioxidants.^84^

### 4.1 Hypertension

Hypertension is one of the major risk factors for AF with reported prevalence rates in AF studies ranging from 49% to 90%.^7^, ^14^ Results from the Atherosclerosis Risk in Communities (ARIC) study suggested that hypertension accounted for approximately 22% of incident AF.^35^ Although there is a strong association between hypertension and AF, an optimal blood pressure target is still subject to debate.^14^ The LIFE (Losartan Intervention For Endpoint reduction in hypertension) study examined whether lower achieved systolic blood pressure (≤130 mm Hg) was associated with a lower incidence of AF, compared to less‐adequate control (systolic blood pressure ≥142 mm Hg) in hypertensive patients suffering from left ventricular hypertrophy.^36^, ^37^ Patients who achieved optimal systolic blood pressure (≤130 mm Hg) had a 40% lower risk of incident AF compared to those with systolic blood pressure ≥142 mm Hg.^7^, ^36^ Another trial by Vermond and colleagues revealed, that the risk of incident AF (HR 1.11) increases with every 10 mm Hg increase in systolic blood pressure.^15^

In light of these findings, it is noteworthy that plant‐based nutrition can lead to a significant decrease in mean blood pressure (BP).^38^ A meta‐analysis by Yokoyama et al found that consumption of vegetarian diets was associated with lower mean systolic BP (−6.9 mm Hg; 95% CI, −9.1 to −4.7; *P* < .001) and diastolic BP (−4.7 mm Hg; 95% CI, −6.3 to −3.1; *P* < .001) in comparison with the consumption of omnivorous diets.^39^ Moreover, the same meta‐analysis revealed that clinical studies of vegetarian or vegan diets of at least 6 weeks duration lead to a mean decrease of 4.8 mm Hg in systolic BP.^39^, ^40^

Plant‐based diets may play an important role not only in the treatment of hypertension but also in its prevention.^38^ A cross‐sectional analysis of 11 004 British women and men comparing several diet groups (meat eaters, fish eaters, vegetarians, and vegans) found the lowest prevalence of hypertension in the vegan group.^41^ Mechanisms by which a plant‐based diet may lead to a decrease in BP include improved vasodilation,^38^, ^42^, ^43^ anti‐inflammatory effects, and a greater serum antioxidant capacity,^38^, ^44^, ^45^ increased potassium intake,^40^, ^46^ modification of the renin‐angiotensin system, and decreased blood viscosity.^38^, ^47^ Reviewing these findings and the association between hypertension and AF, it appears prudent to recommend a plant‐based diet to patients suffering from either condition.

### 4.2 Diabetes and obesity

Two other major risk factors independently associated with AF include obesity and diabetes.^7^, ^48^ Findings from the ARIC study revealed that obesity and overweight (defined as BMI ≥ 25 kg/m^2^) accounted for approximately 18% of incident AF, while 3% of incident AF was attributable to diabetes.^7^, ^35^ Poor glycemic control and longer duration of diabetes are both associated with an increased risk of AF.^49^ A meta‐analysis by Huxley and colleagues revealed a nearly 40% greater risk of incident AF in individuals suffering from diabetes when compared to unaffected patients.^50^ In this context, plant‐based nutrition may also play a key role.

Results from the Adventist Health Study‐2 showed that the prevalence of type 2 diabetes (T2D) among individuals consuming vegetarian diets was shown to be approximately half of that in those following an omnivorous dietary pattern.^51^, ^52^ In fact, vegetarian dietary patterns have not only been associated with lower prevalence and incidence of diabetes, but also with lower body mass index and lower prevalence of metabolic syndrome and its component factors.^53^ A meta‐analysis by Yokoyama and colleagues reviewing clinical trials of vegetarian diets in the treatment of T2D found a significant reduction in HbA1c of −0.39 points compared to control diets.^51^, ^54^

More recent studies published after the release of this meta‐analysis revealed comparable results. In 2016, Lee et al reported the effects of a brown rice‐based vegan diet on glycemic control in patients suffering from T2D and noted a statistically significant HbA1c reduction of −0.9% in participants with high compliance.^55^ In 2017, Ramal and colleagues paired a plant‐based diet low in fat and high in fiber with lifestyle support in the treatment of Latinos with T2D who live in medically underserved areas.^56^ After 6 months, HbA1c levels decreased significantly from 8.53% to 7.31%. Proposed mechanisms by which plant‐based nutrition improves glycemic control and reduces insulin resistance include low content of advanced glycation endproducts, nitrosamines, and a low saturated fat proportion.^54^, ^57^, ^58^ The latter has been shown to significantly contribute to lipotoxicity, a phenomenon in which toxic fat metabolites accumulate in skeletal muscle and hepatic cells, impairing insulin signaling, and thus decreasing glucose uptake.^54^, ^58^

In addition to that, plant‐based diets have frequently been linked to healthier weights.^59^, ^60^, ^61^ A review of long‐term studies in vegans and vegetarians has shown a lower prevalence of overweight and obesity compared with nonvegetarians and vegans from a similar background.^62^, ^63^ According to Appleby and Key, vegetarians usually have a lower BMI than otherwise comparable nonvegetarians, with differences typically in the range of 1‐2 kg/m^2^ across all adult age groups.^62^ Plant‐based diets may not only prevent overweight and obesity, but also promote weight loss.^64^ These findings are important because incremental increases in BMI have been associated with a significant excess risk of AF in various clinical settings.^65^ According to a meta‐analysis by Wong and colleagues, every 5 unit increase in BMI was significantly associated with a 10% increase in postoperative AF.^14^, ^65^ Then again, weight loss has been associated with an improvement in AF burden and severity in short‐ and long‐term studies.^33^, ^66^ In patients suffering from AF, weight loss ≥10% resulted in a sixfold greater probability of arrhythmia‐free survival compared to patients who lost less than 10% of their body weight during the same period.^33^ The study also revealed a dose‐response effect, with arrhythmia‐free survival rates of 40% with ≤3% weight loss, 66% with 3%‐10% weight loss, and 86% with ≥10% weight loss.^33^, ^67^

To achieve and maintain normal weight, adoption of a plant‐based diet might be a useful tool. There is mounting evidence that a plant‐based diet is beneficial in the treatment of T2D and poor metabolic control, which are both established risk factors contributing to an increased risk of AF.^54^, ^68^ Thus, a plant‐based diet might be a key component to achieve the treatment goal of a HbA1c value of ≤7% as suggested by the 2016 European Guidelines on cardiovascular disease prevention in clinical practice.^69^

### 4.3 Inflammation

Moreover, there is emerging evidence pointing at a close relationship between inflammation and AF.^70^ Inflammation may contribute to both the occurrence and maintenance of AF and is suggested to be linked to various pathological processes including oxidative stress, altered calcium homeostasis, cardiomyocyte apoptosis, and ultimately fibrosis, which, in turn, promotes AF substrate formation.^70^, ^71^, ^72^ It is particularly noteworthy that the presence of inflammation in the systemic circulation or the heart can predict the onset of AF and its recurrence in the general population.^71^ Mediators of the inflammatory response can alter atrial electrophysiology and thereby predispose individuals to an increased vulnerability to AF.^71^

A plant‐based diet is rich in anti‐inflammatory and antioxidative components and has been shown to significantly reduce adverse markers of poor cardiovascular health over time.^43^, ^73^, ^74^ Dietary patterns that are rich in unrefined plant foods have been demonstrated to reduce CRP levels in various studies.^40^, ^75^ A study from 2017 found significantly higher CRP levels in omnivores than in vegans (1.1 mg/L vs 0.5 mg/L; *P*‐level: 0.007).^76^ Furthermore, a more recent meta‐analysis investigating the association between vegetarian diets and inflammatory biomarkers found that a vegetarian diet might be a useful approach to manage inflammation in the long term.^77^

Reviewing these findings, the antioxidative and anti‐inflammatory effects of a plant‐based diet might be useful to reduce systemic inflammation, which has been found to contribute to both the occurrence and maintenance of AF.^70^

### 4.4 Heart failure and coronary artery disease

Other important risk factors for AF include heart failure and coronary artery disease (CAD).^7^ Plant‐based diets may beneficially affect both entities and have been shown to be able to halt and even reverse CAD.^78^, ^79^, ^80^ The Lifestyle Heart Trial published by Ornish and colleagues found that more than 80% of patients diagnosed with heart disease who followed a plant‐based diet (lifestyle‐)program had some level of regression of atherosclerosis and 91% had a reduction in the frequency of angina episodes.^81^ In his book “Prevent and reverse heart disease,” Esselstyn demonstrated that switching patients to a plant‐based diet may result in significant (angiographic) disease reversal.^82^ A second larger study including almost 200 patients suffering from significant CAD was published in 2014 by the same author.^83^ About 198 participants were counseled in plant‐based nutrition and asked to consequently eliminate all dairy products, fish, and meat as well as added oils from their daily menu. During the 4 year follow‐up period, 99.4% of the participants who adhered to the whole‐food plant‐based diet avoided any major cardiac event. Angina resolved or improved in almost 93% of participants. A review by Tuso et al identified several mechanisms how a plant‐based diet could prevent CAD events.^84^ These mechanisms include prevention of vascular endothelial cell injury and prevention of LDL oxidation as well as oxidative inflammation by increased intake of antioxidants.^78^, ^84^ Furthermore, prevention of macrophage activation may play an important role.

Heart failure is associated with high rates of morbidity and mortality and, above that, one of the most important risk factors for incident AF.^7^, ^79^ There is interventional and observational evidence that a plant‐based diet may decrease the incidence and severity of heart failure.^80^ More recently, several case reports on plant‐based diets as a potential therapeutic approach in the treatment of heart failure have been published.^85^, ^86^ While well‐designed and large randomized, controlled nutrition intervention trials are missing with regard to this entity, a series of potential mechanisms on how a plant‐based diet may beneficially affect heart failure have been postulated.^80^ These include decreased oxidative stress and inflammation levels, increased nitric oxide bioavailability, reduced homocysteine levels, and increased levels of antioxidants.^80^, ^85^

## 5 DISCUSSION

Atrial fibrillation is the most common arrhythmia and, given its significant burden of morbidity and mortality, a major global public health problem of our time.^1^, ^2^ Targeting risk factors and other underlying conditions that predispose individuals to AF is an emerging management concept that warrants better implementation in daily clinical care.^7^ According to Lau et al, risk factor management is “an essential fourth pillar of AF care” alongside rhythm and rate control as well as appropriate anticoagulation.^87^ Several independent risk factors, including cardiovascular disease such as hypertension, congestive heart failure, valvular heart disease, coronary artery disease, and obesity and diabetes mellitus have been identified as independent predictors of AF development.^7^, ^14^, ^87^ While the authors of this review believe that it is hard to determine which is the strongest or most important risk factor, it is likely that all these predisposing conditions might complementarily contribute to an increased risk of AF. It is now widely accepted that a high cardiovascular risk profile, which is closely related to a sedentary Western lifestyle, is detrimental and requires sustainable management.

In light of these findings, dietary modifications are of paramount importance. Plant‐based diets may reduce the likelihood of many traditional AF risk factors, including hypertension, coronary artery disease, obesity, and diabetes (see Table 1). Moreover, plant‐based nutrition may also play an important role in modifying emerging AF risk factors, such as inflammation.^43^, ^77^

Plant‐based diets emphasize whole grains, legumes, vegetables, fruits, nuts, and seeds while excluding most (or all) animal products. Diets based on whole plant foods not only maximize protective foods, but also exclude potentially harmful animal foods that are high in saturated fat.^54^ As such, avoiding dark fish and limiting caffeine and alcohol intake might be beneficial in patients suffering from AF. The high magnesium content of a whole‐food plant‐based diet is also noteworthy,^88^, ^89^, ^90^ because low serum magnesium is moderately associated with the development of AF in individuals without cardiovascular disease.^29^, ^30^

As postulated by McMacken and Shah, plant‐based diets “address the bigger picture for patients [...]” by simultaneously treating lifestyle diseases (such as diabetes and obesity) and cardiovascular disease.^54^ In this review, we presented evidence for a plant‐based diet beneficially affecting the most common and important AF risk factors.

Ultimately, the reservation must be made that large and randomized controlled trials specifically investigating the effect of a plant‐based diet on AF are missing. Many clinical and epidemiological studies investigated the effects of a plant‐based diet on risk factors predisposing individuals to AF, however, a large trial specifically tailored to patients suffering from AF is missing. Nevertheless, the current literature points at strong evidence for the beneficial effects of plant‐based diets in cardiovascular disease and its risk factors. Physicians halted and reversed coronary artery disease and even heart failure in severely impaired individuals by prescribing a plant‐based diet. Why would not this be possible in patients suffering from atrial fibrillation?

## 6 CONCLUSION

Reviewing the current evidence, a whole‐food plant‐based diet might be a valuable tool in managing and reducing common risk factors that are associated with atrial fibrillation, including hypertension, coronary artery disease, inflammation, obesity, and diabetes. Larger randomized controlled trials investigating plant‐based diets in routine AF management are necessary to confirm those findings.

## CONFLICT OF INTERESTS

The authors received no specific funding for this work. We hereby confirm, that the manuscript has not been accepted elsewhere for publication. The authors declare that there is no conflict of interest. The authors report no proprietary or commercial interest in any product mentioned or concept discussed in this article.
